# Effect of different diastolic blood pressure levels on the prognosis of patients with heart failure after acute myocardial infarction

**DOI:** 10.3389/fcvm.2025.1703466

**Published:** 2026-01-22

**Authors:** Xue Sun, Mengjie Lei, Xiao Wang, Jingyao Wang, Yachao Li, Cairong Li, Zhigang Zhao, Chunyan Zhang, Wanda Ma, Zengming Xue

**Affiliations:** 1 Department of Cardiology, Langfang People’s Hospital, Hebei Medical University, Langfang Core Laboratory of Precision Treatment of CAD, Langfang, China; 2 Graduate School of Hebei North University, Zhangjiakou, China

**Keywords:** acute myocardial infarction, diastolic blood pressure, heart failure, percutaneous coronary intervention, prognosis

## Abstract

**Aims:**

This study aims to investigate the effect of different diastolic blood pressure levels at discharge on the prognosis of patients with heart failure after acute myocardial infarction.

**Methods:**

This study included 642 patients hospitalized in the Department of Cardiology of Langfang People's Hospital who were diagnosed with heart failure after acute myocardial infarction between March 2017 and October 2022. Patients were divided according to diastolic blood pressure (DBP) at discharge into three groups: <70 mmHg (*n* = 122), 70–80 mmHg (*n* = 221), and >80 mmHg (*n* = 299) groups. The follow-up period was 12 months after discharge. The primary endpoint was a composite of all-cause mortality and all-cause readmission during follow-up. Secondary endpoints included the composite endpoint of cardiac death and cardiac readmission, as well as all-cause mortality, cardiac death, all-cause readmission, cardiac readmission, and heart failure-related readmission.

**Results:**

During the follow-up period, there were no significant differences among the three groups in the incidence of the primary endpoint (a composite of all-cause mortality and all-cause readmission) or secondary endpoints (the composite endpoint of cardiac death and cardiac readmission, all-cause mortality, cardiac death, all-cause readmission, cardiac readmission, and heart failure readmission) (*P* > 0.05). Cox regression analysis, adjusted for variables showing differences in the univariate analysis, showed that patients in the 70–80 mmHg group had a significantly higher risk of the primary endpoint than those in the <70 mmHg group (HR: 2.078, 95% CI: 1.009–4.280, *P* = 0.047). Compared with the <70 mmHg group, patients in the >80 mmHg group exhibited an increased risk of the primary endpoint (HR: 2.808, 95% CI: 1.216–6.481, *P* = 0.016), the composite endpoint of cardiac death and cardiac readmission (HR: 3.765, 95% CI: 1.393–10.176, *P* = 0.009), all-cause readmission (HR: 2.850, 95% CI: 1.197–6.789, *P* = 0.018), and cardiac readmission (HR: 3.376, 95% CI: 1.234–9.237, *P* = 0.018), with no significant differences observed for the remaining outcome measures. No significant differences in outcome indices were found between the >80 mmHg and 70–80 mmHg groups (*P* > 0.05).

**Conclusion:**

Different DBP levels at discharge in patients with heart failure after AMI are useful for patient prognosis evaluation. Maybe patients with heart failure after AMI with a low DBP (<70 mmHg) at discharge have a lower risk of all-cause mortality and all-cause readmission. Notably, the study population had a relatively high mean left ventricular ejection fraction, and a higher number of patients in the DBP < 70 mmHg group were treated with MRAs. Since MRAs themselves have blood pressure-lowering effects, their use may have influenced the results and prognosis. Therefore, until these findings are confirmed by further trials, active reduction of diastolic blood pressure should be approached with caution. This conclusion requires validation through large-scale randomized studies.

## Introduction

Although the incidence and severity of ventricular systolic dysfunction and remodeling have been reduced by early coronary revascularization and optimized medical drug therapy after acute myocardial infarction, some patients continues to experience cardiac dysfunction and may progress to heart failure. In addition, myocardial inhibition, inflammation, and coronary microvascular injury contribute to in the development of heart failure (1). Larger infarct size and greater residual myocardial load are associated with a higher possibility of heart failure (2). In the Global Registry of Acute Coronary Events study, the incidence of heart failure at hospital admission was 15.6% among patients with acute ST-elevation myocardial infarction and 15.7% among those with acute non-ST-elevation myocardial infarction (3). Previous studies have shown that heart failure increases mortality in patients with acute coronary syndrome by tenfold (4). Therefore, prognostic management of patients with heart failure following acute myocardial infarction is of particular importance. The 2021 European Society of Cardiology and 2022 American Heart Failure Society guidelines (5) emphasize that, in patients with heart failure, up-titration of beta-blockers or renin–angiotensin–aldosterone system inhibitors to the maximum tolerated or target dose and control of heart rate and blood pressure improve clinical outcomes regardless of the initiation sequence. However, the use of these drugs is not recommended in the presence of hypotension and should be carefully administered and closely monitored when systolic blood pressure is <90 mmHg, or even when approaching the critical value of 100 mmHg. Multiple studies have shown that low blood pressure is associated with poor outcomes in patients with heart failure (6, 7); this association may reflect poor cardiac pump function in patients with heart failure and low blood pressure or because hypotension limits the use of guideline-oriented drugs. To date, most studies have explored the prognostic impact of different systolic blood pressure levels in heart failure, while relatively few have studied the role of different diastolic blood pressure (DBP) levels. A *post hoc* analysis of the ACCORD BP trial found that maintaining diastolic blood pressure within the target range over the long term was independently associated with a reduced risk of primary cardiovascular outcomes (8). The EPHESUS trial (9) showed that a DBP <70 mmHg was associated with increased rates of all-cause mortality, cardiac mortality, and cardiac rehospitalization. However, a *post hoc* analysis revealed no increase in cardiovascular outcome risks associated with low diastolic blood pressure in patients who underwent reperfusion therapy. For patients with heart failure following acute myocardial infarction, the optimal range of diastolic blood pressure associated with the best prognosis remains unclear. This study explored the effect of different DBP levels on the prognosis of patients with acute myocardial infarction complicated by heart failure to provide a clinical reference for blood pressure management and prognosis improvement.

## Methods

### Study population

In this single-center retrospective cohort study, 642 patients with acute myocardial infarction and heart failure who were hospitalized in the Department of Cardiology of Langfang People's Hospital from March 2017 to October 2022 were selected. The average age of the cohort was 60.57 ± 11.19 years. According to the DBP level at discharge, patients were divided into three groups: <70 mmHg (*n* = 122), 70–80 mmHg (*n* = 221), and >80 mmHg (*n* = 299) groups. The inclusion criteria were as follows: patients who met the relevant diagnostic criteria for acute myocardial infarction according to the ESC guidelines and underwent percutaneous coronary intervention; patients who met the relevant diagnostic criteria for heart failure according to the ESC guidelines (10–12); patients aged ≥18 years; and patients who were followed up for ≥12 months. We defined heart failure in accordance with the ESC guidelines (10) and required fulfillment of all of the following criteria: (1) Typical symptoms (e.g., breathlessness, ankle swelling and fatigue) that may be accompanied by signs (e.g., elevated jugular venous pressure, pulmonary crackles and peripheral oedema) caused by a structural and/or functional cardiac abnormality resulting in a reduced cardiac output and/or elevated intracardiac pressures at rest or during stress. Diuretics were administered during hospitalization due to the above situation. (2) Systolic or diastolic left ventricular function impairment confirmed by echocardiography. (3) NT-proBNP ≥ 300 pg/mL. For patients of advanced age, with atrial fibrillation, or with renal insufficiency, NT-proBNP values meet the diagnostic criteria for acute heart failure after correction. The exclusion criteria were as follows: inability to complete follow-up for any reason; diagnosed with malignant tumors, autoimmune diseases, or blood system diseases; estimated glomerular filtration rate <30 mL/min/1.73 m^2^; and severe liver dysfunction.

### Baseline data collection

Baseline clinical data of all enrolled patients were recorded, including age, sex, body mass index (BMI), heart rate at discharge, systolic blood pressure at discharge, smoking history, type of myocardial infarction (acute ST-segment elevation myocardial infarction or acute non-ST-segment myocardial infarction), infarction site, and history of hypertension, diabetes, and other chronic medical histories. Elbow venous blood was collected from patients with an empty stomach in the early morning of the day following admission. White blood cell count, hemoglobin, low-density lipoprotein, uric acid, and other conventional laboratory indicators were measured, and the estimated glomerular filtration rate was calculated. All patients underwent cardiac color ultrasonography on admission, and left ventricular ejection fraction was recorded by the ultrasound department of our hospital. All patients received renin–angiotensin–aldosterone system inhibitors, β-receptor blockers, mineralocorticoid receptor antagonists, and sodium-dependent glucose transporter 2 inhibitors.

Blood pressure of all patients was measured before discharge, and brachial artery blood pressure was measured after at least 5 min of rest. The blood pressure was measured using a manual mercury, automatic, or semi-automatic sphygmomanometer.

### Follow-up and definitions

Trained, full-time data officers conducted the follow-up of patients with heart failure after acute myocardial infarction. Outpatient follow-ups were conducted at 2 weeks, 3 months, 6 months, and 1 year after discharge. Patients who did not attend outpatient follow-up visits were followed up by telephone. We have a standardized heart failure center with professional personnel to conduct outpatient and telephone follow-up of discharged patients. Individualized follow-up monitoring of each patient improves patient compliance with medication. The primary endpoint was a composite of all-cause mortality and all-cause readmission during follow-up. The secondary endpoints included the composite endpoint of cardiac death and cardiac readmission, all-cause mortality, cardiac death, all-cause readmission, cardiac readmission, and heart failure readmission.

All-cause mortality was defined as the ratio of the total number of patients who died from various reasons within 1 year after PCI to the total number of people included in the study.

The causes of cardiac death were as follows: (1) death caused by cardiac shock or heart failure; (2) death caused by complications after acute myocardial infarction, such as ventricular septal perforation, cardiac tamponade, or cardiac rupture; (3) death caused by malignant arrhythmias; and (4) death caused by PCI-related causes.

All-cause readmission was defined as the ratio of the total number of patients admitted for various reasons within 1 year after PCI to the total number of patients included in the study.

Cardiac readmission was defined as the ratio of the number of patients who were readmitted for cardiac-related causes within 1 year after PCI to the total number of patients included in the study.

Heart failure readmission was defined as the ratio of the number of patients who were readmitted for heart failure within 1 year after PCI to the total number of patients included in the study.

### Statistical analysis

Statistical analyses were conducted using SPSS 26.0 software. Data normality was assessed using the Shapiro–Wilk test. Normally distributed data are presented as mean ± standard deviation, and analysis of variance was used for between-groups comparisons. The LSD *t*-test was used to compare the three groups. Non-normally distributed data are presented as median and interquartile range; intergroup comparisons were analyzed using rank sum tests. Count data are expressed as frequencies and percentages, and the chi-square test was used to examine the differences between groups. Sensitivity analyses was performed in the form of subgroup analyses. Cox multivariate regression analysis was used to adjust for baseline data. Differences were considered statistically significant at *P*-values < 0.05.

## Results

### Baseline characteristics

Compared with the 70–80 mmHg group, the <70 mmHg group had a lower prevalence of hypertension, lower systolic blood pressure at discharge, and a higher proportion of patients with a history of smoking. Compared with the >80 mmHg group, the <70 mmHg group had a lower proportion of male patients and patients with hypertension, as well as lower BMI, heart rate at discharge, and systolic blood pressure at discharge, but a higher proportion of patients who underwent emergency PCI and achieved complete RV. The BMI and systolic blood pressure at discharge were lower in the 70–80 mmHg group than in the >80 mmHg group, and the incidence of emergency PCI and complete RV was higher in the 70–80 mmHg group than in the >80 mmHg group (*P* < 0.05). No significant differences were observed for the remaining indices (all *P*’s > 0.05) (Table 1).

**Table 1:** Baseline characteristics.

Characteristics	DBP <70 mmHg	DBP 70–80 mmHg	DBP >80 mmHg	*F*/*χ*²-value	*P*-value
	(*n* = 122)	(*n* = 221)	(*n* = 299)		
Age (years; mean ± SD)	62.62 ± 10.26	60.55 ± 11.68	59.75 ± 11.10	2.877	0.057
Male (*n*, %)	76 (62.30%)	150 (67.87%)	227 (75.92%)^a^	8.915	0.012
BMI (kg/m^2^; mean ± SD)	24.80 ± 3.18	25.61 ± 3.28	26.46 ± 3.23^a^^,^^b^	12.304	<0.001
STEMI (*n*, %)	109 (89.34%)	196 (88.69%)	243 (81.27%)	7.508	0.023
Emergency PCI (*n*, %)	82 (67.21%)	148 (66.97%)	160 (53.51%)^a^^,^^b^	12.291	0.002
Complete RV (*n*, %)	70 (57.38%)	125 (56.56%)	118 (39.47%)^a^^,^^b^	19.349	<0.001
LVEF (%; mean ± SD)	54.51 ± 6.38	54.95 ± 7.27	55.10 ± 7.40	0.298	0.742
Anterior wall series myocardial infarction (*n*, %)	67 (54.92%)	96 (43.44%)	154 (51.51%)	5.158	0.076
Heart rate at discharge (bpm, mean ± SD)	69.31 ± 8.20	71.97 ± 9.79	73.15 ± 10.57^a^	6.546	0.002
Systolic blood pressure at discharge (mmHg, mean ± SD)	106.56 ± 11.42	117.76 ± 12.51^a^	131.82 ± 13.85^a^^,^^b^	184.130	<0.001
Pulse pressure [mmHg, (P25, P75)]	(39.00, 50.00)	(36.50, 52.00)	(40.00, 53.00)	5.668	0.059
Medical history (*n*, %)					
Hypertension	53 (43.44%)	145 (65.61%)^a^	219 (73.24%)^a^	27.001	<0.001
Type 2 diabetes	20 (16.39%)	47 (21.27%)	71 (23.75%)	27.86	0.248
Cerebrovascular disease	11 (9.02%)	19 (8.60%)	46 (15.38%)	6.758	0.034
OMI	6 (4.92%)	12 (5.43%)	22 (7.36%)	1.253	0.535
Atrial fibrillation	5 (4.10%)	5 (2.26%)	9 (3.01%)	0.928	0.629
Current smoker	73 (59.84%)	95 (42.99%)^a^	152 (50.84%)	9.147	0.010
CAD family history	2 (1.64%)	9 (4.07%)	8 (2.68%)	1.777	0.411
Peripheral artery disease	1 (0.82%)	0 (0%)	2 (0.67%)	1.625	0.444
COPD	0 (0%)	0 (0%)	2 (0.67%)	2.301	0.316

DBP, diastolic blood pressure; BMI, body mass index; STEMI, ST-segment elevation myocardial infarction; PCI, percutaneous coronary intervention; RV, revascularization; LVEF, left ventricular ejection fraction; OMI, old myocardial infarction; CAD, coronary artery disease; COPD, chronic obstructive pulmonary disease.

^a^ Compared with the <70 mmHg group, *P* < 0.0167.

^b^ Compared with the 70–80 mmHg group, *P* < 0.0167.

### Laboratory tests

Compared with the >80 mmHg group, the <70 mmHg group exhibited higher levels of NT-proBNP and CK-MB and lower UA levels. The WBC, cTnI, NT-proBNP, and CK-MB levels were higher in the 70–80 mmHg group than in the >80 mmHg group (all *P* < 0.05). No significant differences were observed for the remaining indices (all *P*’s > 0.05) (Table 2).

**Table 2:** Laboratory tests.

Characteristics	DBP <70 mmHg	DBP 70–80 mmHg	DBP >80 mmHg	*F*/*χ²-*value	*P*-value
	(*n* = 122)	(*n* = 221)	(*n* = 299)		
HGB (g/L, mean ± SD)	136.85 ± 17.70	136.43 ± 18.74	139.20 ± 17.81	1.684	0.186
WBC (×10^9^/L, mean ± SD)	8.89 ± 2.95	8.85 ± 2.64	8.25 ± 2.43^b^	4.479	0.012
PLT (×10^9^/L, mean ± SD)	239.04 ± 49.00	241.02 ± 59.34	240.57 ± 59.76	0.048	0.954
FIB (g/L, mean ± SD)	3.52 ± 1.06	3.49 ± 0.93	3.43 ± 0.80	0.629	0.534
cTnI [µg/L, (P25, P75)]	(0.92, 10.11)	(0.89, 11.73)	(0.49, 7.44)^b^	9.469	0.009
NT-proBNP [ng/L, (P25, P75)]	(718.75, 1,907.00)	(663.10, 1,948.00)	(518.00, 1,438.21)^a^^,^^b^	19.088	<0.001
CK-MB [U/L, (P25, P75)]	(10.35, 117.50)	(11.00, 99.50)	(10.00, 61.58)^a^^,^^b^	9.954	0.007
Cr (µmol/L, mean ± SD)	69.17 ± 16.59	67.01 ± 21.13	70.24 ± 19.41	1.750	0.175
eGFR (mL/min/1.73 cm^2^, m ± SD)	91.12 ± 17.02	96.15 ± 20.26	94.18 ± 18.50	2.798	0.062
UA (µmol/L, mean ± SD)	317.34 ± 91.79	327.17 ± 86.90	343.67 ± 96.80^a^	4.174	0.016
FBG (mmol/L, mean ± SD)	6.71 ± 2.60	7.00 ± 2.47	6.64 ± 1.93	1.751	0.175
HbA1C (%, mean ± SD)	6.87 ± 0.68	6.92 ± 1.22	6.97 ± 0.73	0.636	0.530
TC (mmol/L, mean ± SD)	5.11 ± 1.35	4.94 ± 1.36	5.04 ± 1.44	0.659	0.518
TG (mmol/L, mean ± SD)	1.76 ± 1.14	1.84 ± 1.01	1.94 ± 1.19	1.308	0.271
LDL-C (mmol/L, mean ± SD)	2.99 ± 0.91	2.97 ± 0.83	3.00 ± 0.96	0.041	0.960
HDL-C (mmol/L, mean ± SD)	0.89 ± 0.29	0.83 ± 0.30	0.89 ± 0.45	1.940	0.145
Lp(a) (mg/L, mean ± SD)	(187.83, 346.05)	(148.95, 332.73)	(159.40, 332.73)	6,155	0.046

DBP, diastolic blood pressure; HGB, hemoglobin; WBC, white blood cell; PLT, platelet; FIB, fibrinogen; cTnI, cardiac troponin; NT-proBNP, N-terminal B-type natriuretic peptide; CK-MB, creatine kinase-MB; Cr, creatinine; eGFR, estimated glomerular filtration rate; UA, uric acid; FBG, fasting blood glucose; HbA1C, type A1C glycosylated hemoglobin; TC, total cholesterol; TG, triglyceride; LDL-C, low-density lipoprotein cholesterol; HDL-C, high-density lipoprotein cholesterol; Lp(a), lipoprotein(a).

^a^ Compared with the <70 mmHg group, *P* < 0.0167.

^b^ Compared with the 70–80 mmHg group, *P* < 0.0167.

### Medications administered during hospitalization

The proportion of patients administered mineralocorticoid receptor antagonists was higher in the <70 mmHg group than in the >80 mmHg group (*P* < 0.05), and no significant differences were observed for the other indices (all *P*’s > 0.05) (Table 3).

**Table 3:** Medication treatment during hospitalization.

Characteristics	DBP <70 mmHg	DBP 70–80 mmHg	DBP >80 mmHg	F/*χ*^2^*-*value	*P-*value
	(*n* = 122)	(*n* = 221)	(*n* = 299)		
RAAS inhibitors (*n*, %)	38 (31.15%)	69 (31.22%)	95 (31.77%)	0.025	0.988
β-Receptor blockers (*n*, %)	73 (59.84%)	119 (53.85%)	165 (55.18%)	1.183	0.553
MRAs (*n*, %)	59 (48.36%)	84 (38.01%)	89 (29.77%)^a^	13.493	0.001
SGLT2i (*n*, %)	4 (3.28%)	4 (1.81%)	7 (2.34%)	0.743	0.690

DBP, diastolic blood pressure; RAAS, renin–angiotensin–aldosterone system; MRAs, mineralocorticoid receptor antagonist; SGLT2i, sodium-dependent glucose transporter 2 inhibitor.

^a^ Compared with the <70 mmHg group, *P* < 0.0167.

### Endpoints during follow-up

During the follow-up, there were no significant differences among the three groups in the incidence of the primary endpoint (a composite of all-cause mortality and all-cause readmission) or any of the the secondary endpoints (the composite endpoint of cardiac death and cardiac readmission, all-cause mortality, cardiac death, all-cause readmission, cardiac readmission, or heart failure readmission) (*P* > 0.05) (Table 4).

**Table 4:** Endpoints during follow-up.

Characteristics	DBP <70 mmHg	DBP 70–80 mmHg	DBP >80 mmHg	*χ²*-value	*P*-value
	(*n* = 122)	(*n* = 221)	(*n* = 299)		
All-cause mortality and all-cause readmission composite endpoint (*n*, %)	12 (9.84%)	30 (13.57%)	39 (13.04%)	1.089	0.580
Cardiac death and cardiac readmission composite endpoint (*n*, %)	8 (6.56%)	22 (9.95%)	29 (9.70%)	1.261	0.532
All-cause mortality (*n*, %)	1 (0.82%)	6 (2.71%)	2 (0.67%)	4.218	0.121
Cardiac death (*n*, %)	0 (0%)	4 (1.81%)	2 (0.67%)	3.208	0.201
All-cause readmission (*n*, %)	11 (9.02%)	26 (11.76%)	37 (12.37%)	0.977	0.614
Cardiac readmission (*n*, %)	8 (6.56%)	19 (8.60%)	27 (9.03%)	0.703	0.704
Readmission for heart failure (*n*, %)	1 (0.82%)	7 (3.17%)	3 (1%)	4.248	0.120

DBP, diastolic blood pressure.

### Cox multivariate regression results

Variables showing differences in the univariate analysis were included in the regression analysis. These variables were sex, BMI, STEMI, emergency PCI, complete revascularization, heart rate at discharge, systolic blood pressure at discharge, hypertension, cerebrovascular disease, current smoker, WBC, cTnI, NT-proBNP, CK-MB, UA, Lp(a), and ose of MRAs. The results showed that patients in the 70–80 mmHg group had a significantly higher risk of the primary endpoint (HR: 2.078, 95% CI: 1.009–4.280, *P* = 0.047) than those in the <70 mmHg group. Compared with the <70 mmHg group, patients in the >80 mmHg group had an increased risk of the primary endpoint (HR: 2.808, 95% CI: 1.216–6.481, *P* = 0.016), the composite endpoint of cardiac death and cardiac readmission (HR: 3.765, 95% CI:1.393–10.176, *P* = 0.009), all-cause readmission (HR: 2.850, 95% CI: 1.197–6.789, *P* = 0.018), and cardiac readmission (HR: 3.376, 95% CI: 1.234–9.237, *P* = 0.018). No significant differences were observed for the remaining outcome indicators. Outcome indices did not significantly differ between the >80 mmHg and 70–80 mmHg groups (*P* > 0.05) (Table 5).

**Table 5:** Cox regression analysis.

Outcome	Variable	Quotient		Wald	*HR*	95% CI	*P*-value
		*B*	*SE*				
All-cause mortality and all-cause readmission composite endpoint (*n*, %)							
	DBP <70 mmHg					Ref.	–
	DBP 70–80 mmHg	0.731	0.369	3.933	2.078	1.009–4.280	0.047
	DBP >80 mmHg	1.032	0.427	5.850	2.808	1.216–6.481	0.016
Cardiac death and cardiac readmission composite endpoint (*n*, %)							
	DBP < 70 mmHg					Ref.	–
	DBP 70–80 mmHg	0.868	0.443	3.843	2.383	1.000–5.678	0.050
	DBP > 80 mmHg	1.326	0.507	6.826	3.765	1.393–10.176	0.009
All-cause mortality (*n*, %)							
	DBP <70 mmHg					Ref.	–
	DBP 70–80 mmHg	−1.217	1.086	1.256	0.997	0.798–1.245	0.977
	DBP >80 mmHg	0.505	1.229	0.278	1.005	0.813–1.241	0.966
Cardiac death (*n*, %)							
	DBP <70 mmHg					Ref.	–
	DBP 70–80 mmHg	16.572	–	–	1.025	0.821–1.280	0.828
	DBP >80 mmHg	15.265	–	–	1.004	0.814–1.240	0.967
All-cause readmission (*n*, %)							
	DBP < 70 mmHg					Ref.	–
	DBP 70–80 mmHg	0.680	0.389	3.063	1.974	0.922–4.228	0.080
	DBP >80 mmHg	1.047	0.443	5.596	2.850	1.197–6.789	0.018
Cardiac readmission (*n*, %)							
	DBP <70 mmHg					Ref.	–
	DBP 70–80 mmHg	0.711	0.453	2.462	2.036	0.838–4.951	0.117
	DBP >80 mmHg	1.217	0.514	5.612	3.376	1.234–9.237	0.018
Readmission for heart failure (*n*, %)							
	DBP <70 mmHg					Ref.	–
	DBP 70–80 mmHg	0.100	1.075	1.638	1.030	0.824–1.287	0.966
	DBP >80 mmHg	1.376	1.160	0.031	1.005	0.813–1.241	0.795

DBP, diastolic blood pressure.

### Subgroup analysis and sensitivity analysis results

**Figure 1:**
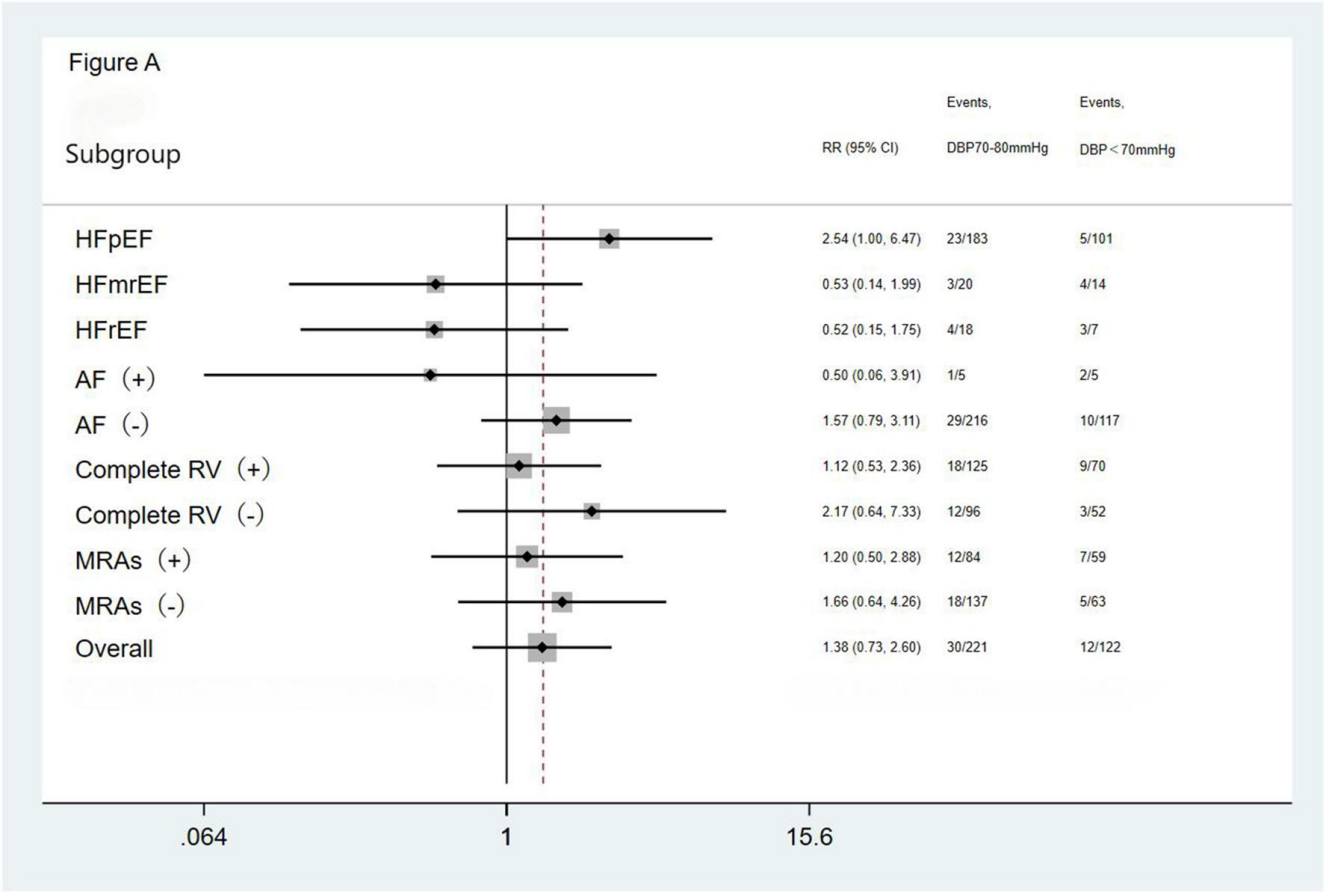
Subgroup analysis of the primary endpoint. HFpEF, heart failure with preserved ejection fraction; HFmrEF, heart failure with mid-range ejection fraction; HFrEF, heart failure with reduced ejection fraction; AF, atrial fibrillation; Complete RV, complete revascularization; MRAs, mineralocorticoid receptor antagonists.

**Figure 2:**
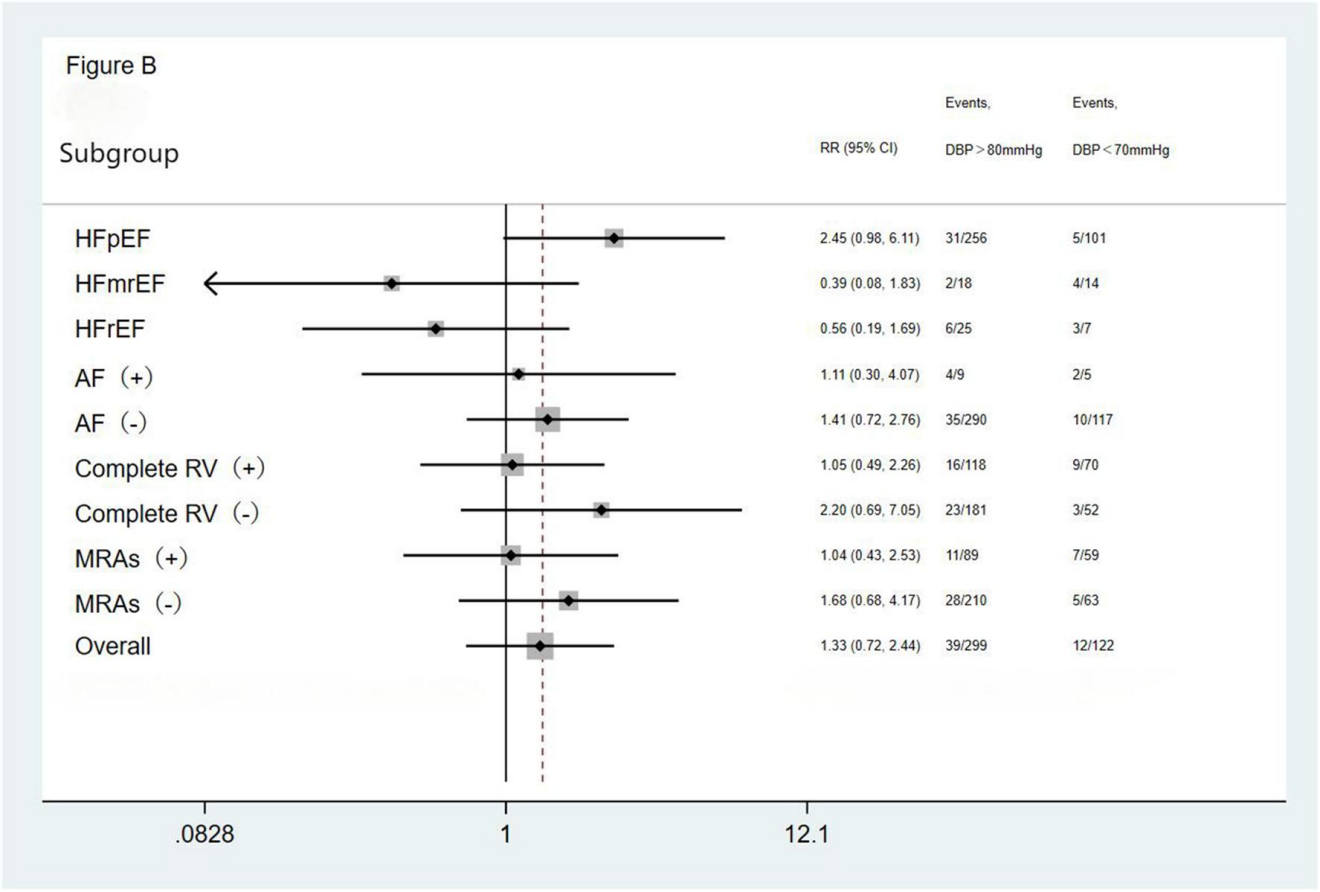
Subgroup analysis of the primary endpoint. HFpEF, heart failure with preserved ejection fraction; HFmrEF, heart failure with mid-range ejection fraction; HFrEF, heart failure with reduced ejection fraction; AF, atrial fibrillation; Complete RV, complete revascularization; MRAs, mineralocorticoid receptor antagonists.

**Figure 3:**
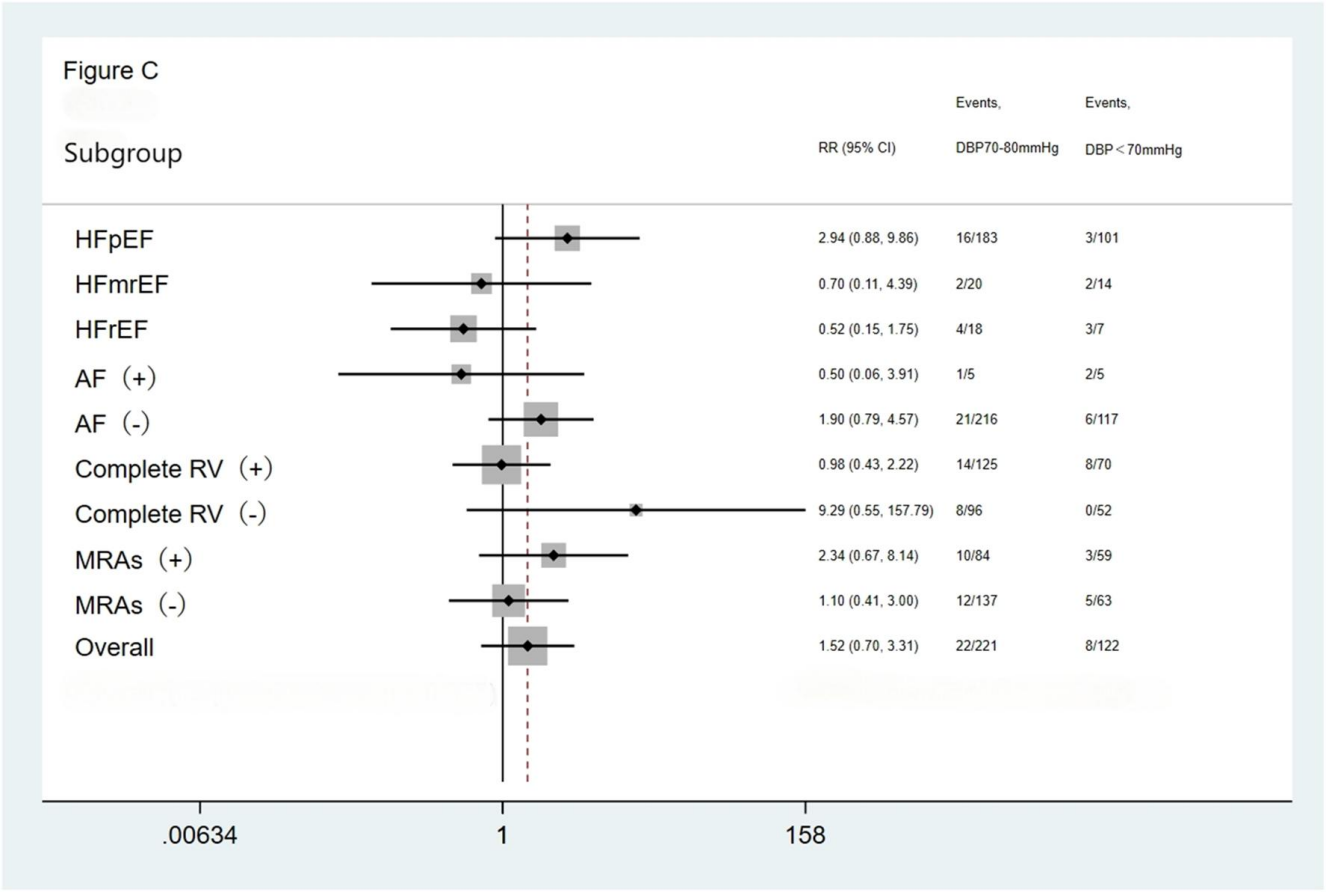
Subgroup analysis of the composite cardiac death and cardiac readmission. HFpEF, heart failure with preserved ejection fraction; HFmrEF, heart failure with mid-range ejection fraction; HFrEF, heart failure with reduced ejection fraction; AF, atrial fibrillation; Complete RV, complete revascularization; MRAs, mineralocorticoid receptor antagonists.

**Figure 4:**
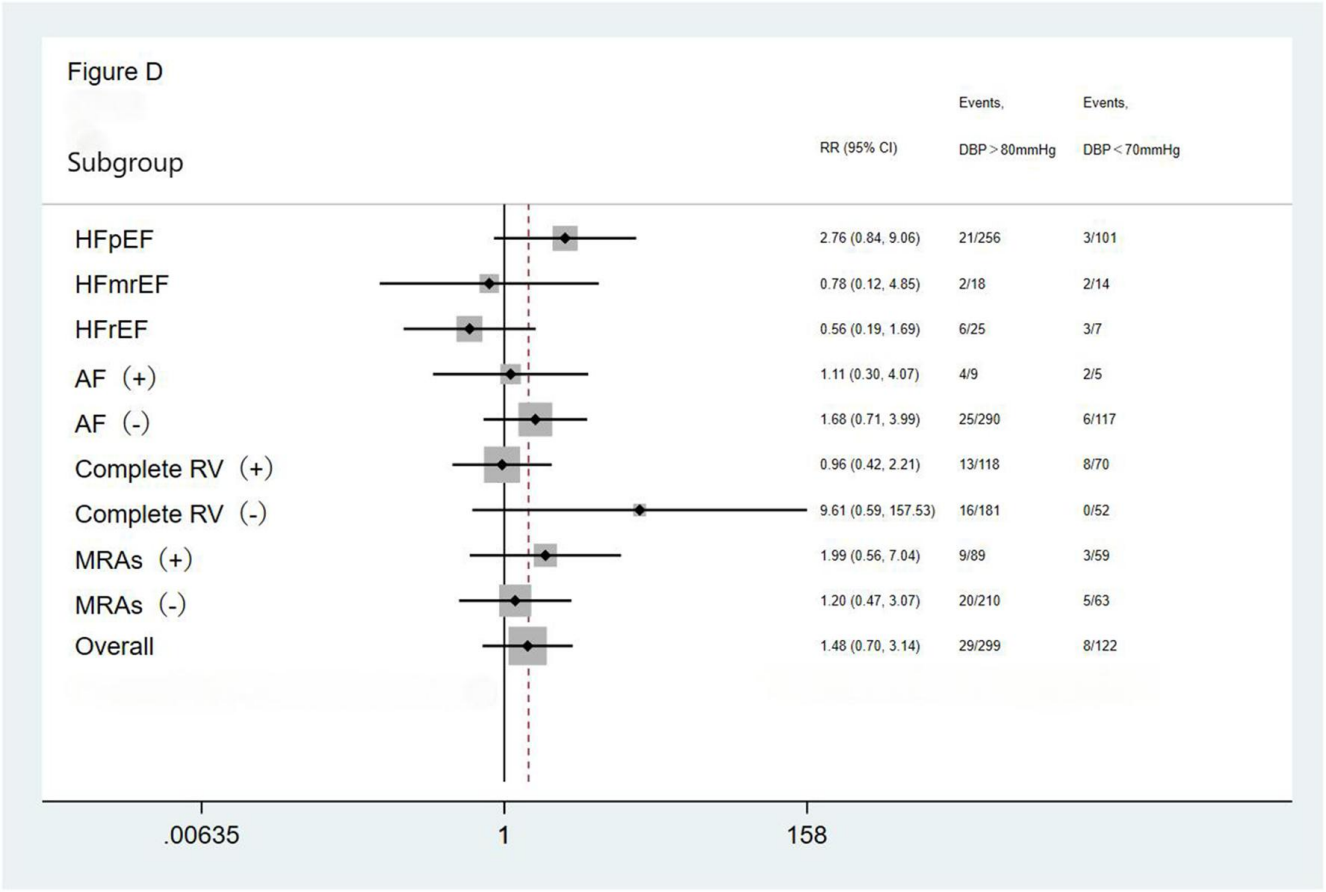
Subgroup analysis of the composite cardiac death and cardiac readmission. HFpEF, heart failure with preserved ejection fraction; HFmrEF, heart failure with mid-range ejection fraction; HFrEF, heart failure with reduced ejection fraction; AF, atrial fibrillation; Complete RV, complete revascularization; MRAs, mineralocorticoid receptor antagonists.

We performed subgroup analysis based on LVEF, the presence of AF, completeness of revascularization, and use of MRAs. The result showed no significant differences in the incidence of the primary endpoint (a composite of all-cause mortality and all-cause readmission) and the composite endpoint of cardiac death and cardiac readmission among the three groups(the <70 mmHg group, the 70–80 mmHg group, and the >80 mmHg group) during the follow-up period. The results showed that the outcomes across all subgroups were consistent with the overall results for both the primary endpoint (a composite of all-cause mortality and all-cause readmission) and the composite endpoint of cardiac death and cardiac readmission during follow-up (Figures 1–4).

## Discussion

Our study is the first to analyze the impact of different DBP levels at discharge on the prognosis of patients with heart failure after acute myocardial infarction. This study retrospectively analyzed the impact of different DBP levels on the prognosis of 642 patients with heart failure after acute myocardial infarction and found that nearly 47% had a DBP >80 mmHg at discharge, 34% had a DBP of 70–80 mmHg, and 19% had a DBP <70 mmHg. During the 12-month follow-up, the incidence of the primary endpoint was significantly higher in the 70–80 mmHg and >80 mmHg groups, at 2.078 and 2.808 times, compared with the <70 mmHg group; similarly, the incidence of the composite cardiac death and cardiac readmission was 2.383 and 3.765 times higher in the 70–80 mmHg and >80 mmHg groups, respectively, compared with that in the <70 mmHg group. The incidence of all-cause and cardiac readmission was 2.850 and 3.376 times higher in the >80 mmHg group, respectively, compared with those in the <70 mmHg group. Therefore, different DBP levels at discharge in patients with heart failure after AMI are useful for patient prognosis evaluation. Maybe patients with heart failure after AMI with a low DBP (<70 mmHg) at discharge have a lower risk of all-cause mortality and all-cause readmission.

Previous studies have found that long-term hypertension leads to impaired cardiac pump function and output, thereby worsening the prognosis of patients with heart failure (13). In patients at higher risk of cardiovascular disease (including clinical or subclinical cardiovascular disease, chronic kidney disease with an estimated glomerular filtration rate of 20–59 mL/min/1.73 m^2^, a Framingham cardiovascular 10-year cardiovascular risk score of >15%, and age ≥75 years) without a history of diabetes or stroke, intensive blood pressure lowering (systolic blood pressure <120 mmHg) is associated with a lower risk of acute coronary syndrome, stroke, acute decompensated heart failure, or cardiac death compared with standard blood pressure lowering (systolic blood pressure <140 mmHg) (14), although guidelines indicate that lowering blood pressure can improve the incidence cardiovascular events and cardiovascular mortality (15, 16). However, aggressive hypotension can also lead to an increase in adverse events, mainly manifested as tissue hypoperfusion, which can negative affect the prognosis of patients. The relationship between blood pressure and cardiovascular events in patients with acute coronary syndrome is J- or U-shaped, and the incidence of cardiovascular events is the lowest when the systolic blood pressure is controlled at 130–140 mmHg and DBP is maintained at 80–90 mmHg (17).

The Korean KorAHF study (18) enrolled 5,625 patients with acute heart failure and calculated their average blood pressure based on measurements taken at discharge and during follow-up, with a median follow-up duration of 2.2 years. Similar to the present study, it also assessed the impact of different diastolic blood pressure levels on all-cause mortality in patients with heart failure. The KorAHF study was the first to propose an inverse J-shaped curve relationship between blood pressure and all-cause mortality in patients with heart failure and also demonstrated that excessively low blood pressure was associated with an increased risk of heart failure readmission. These findings suggests that lower blood pressure in patients with heart failure is not always correlated with improved prognosis. The lowest mortality rate was observed at a systolic/diastolic blood pressure of 132.4/74.2 mmHg, whereas an average systolic/diastolic blood pressure <130/70 mmHg during the discharge and follow-up period was associated with reduced survival in patients with heart failure. This finding differs from the results of our study, which suggest that patients with a discharge diastolic blood pressure <70 mmHg may have a lower risk of all-cause mortality and all-cause readmission. We primarily attribute this difference to the significant disparity in the patient populations enrolled in the two studies. In the KorAHF study, patients with diastolic blood pressure <70 mmHg had an average LVEF of 37%–38%, with HFrEF accounting for 59.4% of cases and HFpEF for only 26.0%. Approximately 9%–11% of patients were classified as NYHA class III/IV, indicating poorer cardiac function. In contrast, the patients in our study exhibited relatively better cardiac function, with an average LVEF ranging from 54.51% to 55.10%, and HFpEF accounting for up to 84% of cases. The enrolled population primarily consisted of patients with mild heart failure. This suggests that, compared to the KorAHF study, the subgroup with diastolic blood pressure <70 mmHg in our study represented a lower-risk population, which likely contributed to the generally better prognosis observed in this subgroup. In additional, previous research has shown that the J-shaped association between blood pressure and mortality is particularly pronounced in elderly individuals or patients with cardiovascular disease (19). In the KorAHF study, the average age of patients with diastolic blood pressure <70 mmHg ranged from 68 to 73 years, indicating an older population. In our study, the average age of this subgroup was 62.62 ± 10.26 years, representing a comparatively younger cohort. Furthermore, compared to the KorAHF study, patients with diastolic blood pressure <70 mmHg in our study had lower prevalences of hypertension, cerebrovascular disease, diabetes, and family history of coronary heart disease, indicating a cardiovascular risk population. These differences may have attenuated the J-shaped association in our study. Another study also supports the notion that, among high-risk patients with heart failure after acute myocardial infarction, those with diastolic blood pressure ≤68, 69–72, and 77–81 mmHg have higher rates of cardiovascular mortality, all-cause mortality, myocardial infarction readmission, and heart failure readmission compared to patients with diastolic blood pressure of 73–76 mmHg (20). Research indicates that low systolic blood pressure (<125 mmHg) is an independent predictor of 1-year endogenous mortality in acute myocardial infarction patients aged 75 and older (21). Elderly individuals may be more prone to hypotension-related adverse cardiovascular events. In contrast, the average age of patients included in this study was relatively younger, and even with a low diastolic blood pressure (<70 mmHg), their risk of adverse cardiovascular events was lower than that observed in the elderly population. In addition, the KorAHF study also reported higher proportions of pulmonary congestion and left bundle branch block in patients with low diastolic blood pressure, further indicating that the subgroup with diastolic blood pressure <70 mmHg represented a higher-risk population. The inclusion of comparisons regarding pulmonary congestion and left bundle branch block proportions is also an area where our study needs to improve and learn. Subgroup analysis results showed that an inverse J-shaped relationship exists for all-cause mortality in both HFrEF and HFpEF patients. Although the association between diastolic blood pressure and increased mortality was more pronounced in patients with heart failure with preserved ejection fraction (HFpEF), the lowest risk point for this group occurred at 127.9/72.7 mmHg, while for HFrEF patients, the lowest risk point occurs at a systolic/diastolic blood pressure of 136.0/76.6 mmHg. In this study, the proportion of HFpEF patients was relatively high, and the optimal diastolic blood pressure was lower, which may also explain why the prognosis is better in the diastolic blood pressure <70 mmHg group in this study. For HFpEF patients, mortality increased significantly when diastolic blood pressure was either too low or too high. However, in HFrEF patients, significantly increased mortality was observed only when the diastolic blood pressure was too low. The higher proportion of HFpEF patients in this study may also lead to a relatively poorer prognosis in the high diastolic blood pressure group, which could further explain why the prognosis is better in the diastolic blood pressure <70 mmHg group in this study. To some extent, the conclusions of our study are not contradictory to those of the KorAHF study. Moreover, both this study and the KorAHF study share the same conclusion that excessively high blood pressure increases cardiovascular risk.

In the present study, compared to the patients with diastolic blood pressure of 70–80 mmHg and >80 mmHg, patients with diastolic blood pressure <70 mmHg exhibited higher proportions of STEMI, emergency PCI, and complete revascularization. Differences in coronary reperfusion status may have influenced outcomes, as improvement in myocardial ischemia following reperfusion can reduce mortality and readmission rates. Early and timely revascularization can enhance patient prognosis. In this regard, our findings are consistent with a *post hoc* analysis of the EPHESUS study, which demonstrated that myocardial reperfusion therapy can reverse the J-shaped relationship between cardiovascular risk and diastolic blood pressure in patients with left ventricular dysfunction and heart failure following myocardial infarction. Among patients who did not receive reperfusion therapy, a diastolic blood pressure <70 mmHg was associated with increased risks of all-cause mortality (HR: 1.8, 95% CI: 1.41–2.30, *P* < 0.001), cardiovascular mortality (HR: 1.70, 95% CI: 1.3–3.22, *P* < 0.001), and cardiac readmission (HR: 1.54, 95% CI: 1.26–1.87, *P* < 0.001). However, in patients who underwent reperfusion therapy, low diastolic blood pressure was not associated with an increased risk of adverse cardiovascular outcomes. In the EPHESUS study, compared to other subgroups, the subgroup with diastolic blood pressure (DBP) <70 mmHg had a lower left ventricular ejection fraction (LVEF). Similarly, in our study, the DBP <70 mmHg group exhibited a lower LVEF relative to other groups. However, when comparing the DBP <70 mmHg groups specifically, the average LVEF in the EPHESUS study was significantly lower than that observed in our study (31.5% ± 6.7% vs. 54.51% ± 6.38%). In the EPHESUS study, approximately 20% of patients were classified as Killip class III/IV, with a mean age of 65.3 ± 12.2 years. Considering factors such as disease severity and age, the population enrolled in our study was at a lower overall risk of disease progression. This difference may explain why, in our DBP <70 mmHg group, we not only did not observe an increased risk of adverse cardiovascular outcomes but also found an improvement in the composite endpoint of all-cause mortality and all-cause readmission. The EPHESUS study found that among patients who underwent reperfusion therapy, low DBP was not associated with an increased risk of adverse cardiovascular outcomes. Furthermore, the reduction in cardiovascular mortality and cardiovascular hospitalization associated with reperfusion therapy was more pronounced in patients with DBP <70 mmHg compared to those who did not receive reperfusion therapy. Combined with the findings of this study, the higher proportion of patients undergoing reperfusion therapy and the better prognosis observed in the diastolic blood pressure <70 mmHg subgroup further indicate that the requirement for adequate diastolic pressure may be more pronounced during ischemic conditions (9). Coronary blood flow depends on the degree of coronary stenosis and coronary perfusion pressure. Severe coronary stenosis can affect coronary blood flow, and approximately 85% of myocardial perfusion occurs during diastole; therefore, diastolic blood pressure plays a very important role in myocardial perfusion (22). However, for patients undergoing reperfusion treatment, the effect of low diastolic blood pressure on poor prognosis is not obvious. This may be due to the improvement in coronary blood supply after coronary vessel opening, which attenuates the effect of low diastolic blood pressure on coronary blood perfusion to some extent. In addition, perfusion pressures as low as 35 mmHg were observed in healthy dogs to maintain myocardial perfusion; however, this threshold may be altered in the presence of coronary inflammation or microvascular disease (23). Attenuation of coronary inflammation after reperfusion treatment may mitigate the prognostic adverse effects. Meanwhile, the INVEST study reported no evidence of a J-shaped relationship between low DBP and poor prognosis in populations without obstructive coronary artery disease (24). After reperfusion, low DBP had no significant effect on poor prognosis.

Maintaining an appropriate level of diastolic blood pressure has received increasing clinical attention. A *post hoc* analysis of the ACCORD BP trial examined the prognostic implications of maintaining optimal diastolic blood pressure levels for various durations. The study found that among hypertensive patients with diabetes, a longer duration of maintaining diastolic blood pressure within the range of 70–80 mmHg was associated with a lower risk of the primary outcome (a composite of first occurrence of non-fatal myocardial infarction, non-fatal stroke, or cardiovascular death) and a reduced risk of non-fatal myocardial infarction. Under conditions of well-controlled systolic blood pressure, maintaining diastolic pressure within an optimal target range may reduce the risk of myocardial infarction (8), providing further evidence of the significant clinical relevance of diastolic blood pressure for prognosis.

We found that the mean ejection fraction of the study population was approximately 55%. The reason may be that the population we included comprised patients with heart failure after acute myocardial infarction rather than patients with pre-existing chronic heart failure. Some of the included patients had heart failure with preserved ejection fraction and also met the diagnostic criteria for heart failure based on the presence of symptoms and signs of heart failure or elevated laboratory indicators such as NT-proBNP. All patients included in this patient underwent inpatient PCI. Coronary revascularization and the early, standardized use of acute myocardial infarction and heart failure drugs during hospitalization may have delayed the decrease in LVEF. At the same time, previous studies have reported that the mean ejection fraction in patients with heart failure after acute myocardial infarction is 54.5 or even higher (25, 26). Prior research has shown that among patients with acute myocardial infarction and an LVEF <50%, a systolic depression (90–99 mmHg) is associated with increased in-hospital cardiovascular mortality. In patients with acute myocardial infarction and an LVEF >50%, elevated systolic blood pressure (>140 mmHg) has been associated with an increase in long-term cardiovascular mortality (27).

Currently, the relationship between diastolic hypertension and cardiovascular risk remains controversial, especially among elderly individuals. With increasing age, the association between diastolic blood pressure and cardiovascular risk gradually diminishes (28, 29). Previous studies suggest that increased arterial stiffness and reduced elasticity in the elderly may lead to lower diastolic blood pressure, which potentially explains the lack of a significant association between diastolic hypertension and cardiovascular risk in this population (30, 31). Research has also shown that among elderly individuals with systolic blood pressure below 130 mmHg, a diastolic blood pressure of 80–90 mmHg does not significantly increase the risk of cardiovascular organ damage or mortality compared to those with diastolic blood pressure <80 mmHg (32). In this study, no significant differences in age were observed among the three patient groups, which may mitigate the interference of the diminished impact of diastolic blood pressure in the elderly, thereby further supporting the robustness of our research findings. Meanwhile, with aging, reduced arterial elasticity and increased stiffness of large arteries tend to result in elevated systolic blood pressure and lower diastolic blood pressure. A lower diastolic blood pressure often corresponds to a higher pulse pressure, and studies have found that an increased pulse pressure has been associated with elevated cardiovascular risk (33). Our study found no significant differences in pulse pressure among the <70 mmHg, 70–80 mmHg, and >80 mmHg diastolic blood pressure groups, further supporting the robustness of our research findings.

This study has several limitations. First, blood pressure may fluctuate over time, and a single measurement obtained at discharge may not truly reflect the 24-h blood pressure profile of patients. DBP was measured only at discharge, without accounting for longitudinal fluctuations. In future research, we will also include data on blood pressure measurements obtained during follow-up. Averaging multiple measurements during hospitalization and follow-up may provide a more objective assessment. Second, due to the limited sample size, we categorized patients with a DBP <70 mmHg as the hypodiastolic group, 70–80 mmHg as the hyperdiastolic group, and >80 mmHg as the hyperdiastolic group, which resulted in wide blood pressure intervals. The lower limit of the <70 mmHg group and the upper limit of the >80 mmHg group were not clearly defined. Broad DBP categories may obscure nuanced relationships. The subdivision may be more representative, which is also a weakness of this study. In future studies, we will refine DBP categories to assess thresholds more precisely. Third, the mean left ventricular ejection fraction of the study population was approximately 55%, with a relatively high proportion of HFpEF. This suggests that the included population may not fully represent the characteristics of all patients with heart failure after acute myocardial infarction (34), potentially limiting the generalizability of the final results. Future multicenter, prospective, randomized studies are needed to further validate the conclusions we have drawn. Fourth, our study intends to explore the effect of different diastolic blood pressure levels on the outcomes in patients with heart failure after acute myocardial infarction. However, while some critically ill patients may have died during hospitalization, this introduces potential survivor bias, meaning that the study population may not fully represent the overall characteristics of patients with heart failure after acute myocardial infarction. The follow-up time of our study was limited to 12 months after discharge. Despite the findings of this study, several limitations remain, and further confirmation through long-term follow-up results is needed. Fifth, this was a single-center study with a small sample size. As mentioned above, we did not stratify the groups into smaller DBP ranges, and differences in age and range of myocardial infarction may also affect the final results. Sixth, in the univariate analysis, we did not detect significant differences in the incidence of primary and secondary endpoints among the three groups during the follow-up period. In the survival analysis, we did not identify any significant time-dependent effect of different DBP levels on prognoses. Future studies should expand the follow-up period and sample size.

## Conclusion

Different DBP levels at discharge in patients with heart failure after AMI are useful for patient prognosis evaluation. Maybe patients with heart failure after AMI with a low DBP (<70 mmHg) at discharge have a lower risk of all-cause mortality and all-cause readmission. The study population had a relatively high mean left ventricular ejection fraction, and a greater number of patients in the group with DBP < 70 mmHg were treated with MRAs. Since MRAs themselves have blood pressure-lowering effects, their use may have influenced the results and prognosis. Therefore, until these findings are confirmed by further trials, active reduction of diastolic blood pressure should be approached with caution. This conclusion requires validation through large-scale randomized studies.

## Data Availability

The original contributions presented in the study are included in the article/Supplementary Material, further inquiries can be directed to the corresponding author.
